# Mannose-Binding Lectin Blunts Macrophage Polarization and Ameliorates Lupus Nephritis

**DOI:** 10.1371/journal.pone.0062465

**Published:** 2013-04-23

**Authors:** Yanxing Cai, Weijuan Zhang, Sidong Xiong

**Affiliations:** 1 Department of Immunology and Institute for Immunobiology, Shanghai Medical College, Fudan University, Shanghai, People’s Republic of China; 2 Institutes of Biology and Medical Sciences, Soochow University, Suzhou, People’s Republic of China; Northwestern University Feinberg School of Medicine, United States of America

## Abstract

**Background:**

Deficiency in clearance of self nuclear antigens, including DNA, is the hallmark of systemic lupus erythematosus (SLE), a chronic autoimmnue disease characterized by the production of various autoantibodies, immune complex deposition and severe organ damage. Our previous studies revealed that administration of syngeneic BALB/c mice with activated lymphocyte-derived DNA (ALD-DNA) could induce SLE disease. Mannose-binding lectin (MBL), a secreted pattern recognition receptor with binding activity to DNA, has been proved to be a modulator of inflammation, but whether MBL takes responsibility for DNA clearance, modulates the DNA-mediated immune responses, and is involved in the development of DNA-induced SLE disease remain poorly understood.

**Methodology/Principal Findings:**

The levels of serum MBL significantly decreased in lupus mice induced by ALD-DNA and were negatively correlated with SLE disease. MBL blunted macrophage M2b polarization by inhibiting the MAPK and NF-κB signaling while enhancing the activation of CREB. Furthermore, MBL suppressed the ability of ALD-DNA–stimulated macrophages to polarize T cells toward Th1 cells and Th17 cells. Importantly, MBL supplement *in vivo* could ameliorate lupus nephritis.

**Conclusion/Significance:**

These results suggest MBL supplement could alleviate SLE disease and might imply a potential therapeutic strategy for DNA-induced SLE, which would further our understanding of the protective role of MBL in SLE disease.

## Introduction

Systemic lupus erythematosus (SLE) is a chronic autoimmune disease characterized by the production of various autoantibodes, complement activation, immune complex (IC) deposition and the subsequent inflammation that contribute to severe organ damage [1], [2]. The precise etiology of SLE remains elusive; however, inefficient clearance of self nuclear antigens released from apoptotic cells has been implicated as an important factor leading to the initiation and development of SLE [3]–[5]. Accumulation of self nuclear antigens, including DNA and RNA, would trigger the autoimmune responses that eventually initiate the production of autoantibodies in SLE patients.

In our previous studies, a novel murine model of SLE was generated by immunizing the syngeneic female BALB/c mice with activated lymphocyte-derived DNA (ALD-DNA), which developed high titers of anti-dsDNA antibodies, IC deposition, proteinuria, and glomerular nephritis that closely resembled human SLE [6]–[8]. We further found that ALD-DNA could induce macrophage M2b polarization [9], which was consistent with previous reports on macrophage M2b polarization in lupus nephritis [10]. These findings suggest that ALD-DNA might serve as an important autoimmunogen to initiate the autoimmune responses that eventually lead to the pathogenesis of SLE. Therefore, recognition and elimination of the autoimmunogen such as ALD-DNA is essential to prevent and treat DNA-induced SLE and other autoimmune diseases.

During lymphocyte activation induced by infection, stress, and other danger signals, DNA was released from activated lymphocytes, but not always provoking the autoimmunity, indicating that free DNA could be removed by the intrinsic physiological mechanisms [11]. Clearance of DNA is important for maintaining immune homeostasis and preventing SLE disease. Therefore, it is essential to study the intrinsic physiological mechanisms of recognizing and eliminating DNA.

The complement system, which is one of the first defence in the innate immunity, is important for recognizing and eliminating invading microorganisms and clearance of cellular debris, apoptotic cells and immune complexes to maintain tissue homeostasis [12]–[14]. Inherited deficiency of complement components has been reported to be associated with the development of autoimmune diseases [15]. Mannose-binding lectin (MBL), one member of complement components, is a secreted pattern recognition receptor (PRR) and mainly produced by the liver during the acute phase response at early stages of infection [16]–[18]. In addition to the binding ability of MBL to the carbohydrates of microorganisms, accumulating data indicate that MBL can enhance the uptake of apoptotic cells and immune complexes by the interaction with its receptors on the cell surface, such as C1q phagocytic receptor C1qRp (CD93), cC1qR (CRT) and CR1 (CD35), which might play a protective role in the development of autoimmune diseases [19]–[25]. It has been reported that MBL deficiency or low serum MBL levels have been observed in SLE [26]–[28]. Previous studies indicate that MBL has the ability of recognizing DNA and is a modulator of inflammatory responses, but whether MBL takes responsibility for DNA clearance, modulates the DNA-mediated immune responses, and plays a protective role in DNA-induced SLE disease remain poorly understood [29]–[32].

In this present study, we examined the levels of serum MBL and found that they decreased in ALD-DNA–induced lupus mice and were negatively correlated with the levels of anti-dsDNA antibodies and urine protein in SLE mice. Furthermore, MBL could blunt ALD-DNA–induced macrophage M2b polarization by down-regulating the MAPK and NF-κB signaling while up-regulating CREB activation and suppress the ability of ALD-DNA–stimulated macrophages to promote T cell differentiation. MBL supplement *in vivo* could ameliorate ALD-DNA–induced lupus nephritis by decreasing anti-dsDNA antibodies production and IC deposition. These results might provide MBL as a potential therapeutic strategy for DNA-induced SLE and other autoimmune diseases.

## Materials and Methods

### Ethics Statement

All experiments carried out in this study were strictly performed in a manner to minimize suffering of laboratory mice. All animal procedures were performed according to the Guide for the Care and Use of Medical Laboratory Animals (Ministry of Health, P.R. China, 1998) and with the ethical approval of the Shanghai Medical Laboratory Animal Care and Use Committee (Permit number: SYXK 2010–0020) as well as the Ethical Committee of Fudan University (Permit number: 2010015).

### Mice and Cells

Six-week-old female BALB/c mice were purchased from the Experimental Animal Center of Chinese Academy of Sciences (Shanghai, P. R. China) and housed in a pathogen-free environment. RAW264.7 cells were purchased from Chinese Academy of Sciences (Shanghai, P. R. China). Peritoneal macrophages were collected as described previously [33].

### Plasmid and Purification of Mouse MBL

The full length of MBL cDNA was amplified from total RNA of murine liver using the primers 5′-ATG CTT CTG CTT CCA TTA CTC CCT GT-3′ and 5′-GGC TGG GAA CTC GCA GAC AGC C-3′. MBL cDNA was inserted into the pcDNA3.1 vector (Invitrogen) to generate pcDNA3.1-MBL plasmid (pMBL). The plasmid construct was confirmed by DNA sequencing. 293T cells were transfected with pMBL plasmid using Lipofectamine 2000 transfection reagent. 4 days after transfection, the supernatants were collected for purification of mouse MBL protein. The purification of mouse MBL protein was performed as described previously [34].

### DNA Preparation

ALD-DNA and UnALD-DNA were prepared with murine splenocytes which were generated from surgical resected spleens of six- to eight-week-old female BALB/c mice and cultured with or without Con A (Sigma-Aldrich) *in vitro* as previously described [7]. Briefly, for generation of ALD-DNA, splenocytes were seeded at 2×10^6^ cells/ml in 75 cm^2^/cell culture flask and cultured in the presence of Con A (5 µg/ml) for 6 days to induce apoptosis. The apoptotic cells were stained with FITC-labeled Annexin V (BD Biosciences) and propidium iodide (PI; Sigma-Aldrich), and sorted using a FACSAria (BD Biosciences). Genomic DNAs from syngeneic apoptotic splenocytes were treated with S1 nuclease (TaKaRa) and proteinase K (Sigma-Aldrich), and then purified using the DNeasy Blood & Tissue Kits (Qiagen) according to the manufacturer’s instructions. UnALD-DNA was prepared with unactivated (resting) splenocytes and extracted using the same methods. To exclude contaminations with LPS, sterile endotoxin-free plastic ware and reagents were used for DNA preparation. DNA samples were also monitored for low level of endotoxin by the Limulus amoebocyte lysate assay (BioWhittaker) according to the manufacturer’s instructions. The concentration of DNA was determined by detection of the absorbance (A) at 260 nm. The apoptotic DNA ladder of ALD-DNA was confirmed by agarose gel electrophoresis (AGE).

### Generation of SLE Murine Model

To generate SLE murine model, 6- to 8-week-old syngeneic female BALB/c mice were divided into several groups of 8–10 mice and actively immunized by subcutaneous injection on the back with 0.2 ml of an emulsion containing ALD-DNA (50 µg/mouse) in phosphate-buffered saline (PBS) plus equal volume of complete Freund’s adjuvant (CFA; Sigma-Aldrich) at week 0, and followed by two booster immunizations of ALD-DNA (50 µg/mouse) emulsified with IFA (Sigma-Aldrich) at week 2 and week 4 for total 3 times as previously described [7]. 8–10 mice in each group received an equal volume of PBS plus CFA or IFA, or UnALD-DNA (50 µg/mouse) plus CFA or IFA were used as controls. Mice were bled from retro-orbital sinus prior to immunization and at 2-week internals until 3 months after the initial immunization. 8 or 12 weeks later, mice were sacrificed and surgical resected spleens and kidneys were collected for further cellular function and tissue histology analysis.

### Autoantibody and Proteinuria Examination

Anti-dsDNA antibodies in the mice serum were determined by ELISA assay as described previously [7]. In briefly, ELISA plates (Costar) were pre-treated with protamine sulphate (Sigma-Aldrich) and then coated with calf thymus dsDNA (Sigma-Aldrich). After incubation with mouse serum, the levels of anti-dsDNA Abs were detected with the horseradish peroxidase (HRP)-conjugated goat anti-mouse IgG (Southern Biotech). Tetramethylbenzidine (TMB) substrate was used to develop colors and absorbance at 450 nm was measured on a microplate reader (BIO-TEK ELX800). Proteinuria of the mice was measured with the BCA Protein Assay Kit (Thermo Scientific) according to the manufacturer’s instructions.

### Reagents and Pharmacological Inhibitor Treatment

The pharmacological reagents were obtained from Calbiochem (San Diego, CA) and were reconstituted in sterile DMSO and used at the following concentrations: NF-κB inhibitor PDTC (50 µM), MEK1/2 inhibitor U0126 (10 µM), p38 MAPK inhibitor SB203580 (20 µM), and JNK inhibitor SP600125 (50 µM). DMSO at 0.1% concentration was used as the vehicle control. In all experiments with inhibitors, a tested concentration was used after careful titration experiments assessing the viability of the macrophages, and the chosen concentrations are in agreement with published reports [7]. In addition, when a given inhibitor was tested, its efficacy in terms of inhibition of phosphorylation of the intended signaling molecule, as well as a nonintended signaling molecule, was also tested. In the experiments with inhibitors, the macrophages were treated with a given inhibitor for 1 h before ALD-DNA stimulation.

### Measurement of Circulating DNA level

DNA was extracted from serum samples and then quantified using a PicoGreen DNA detection kit (Invitrogen) according to the manufacturer’s instructions [8]. In briefly, DNA was extracted from 200 µl of serum samples using a QIAamp Blood Kit (Qiagen) using the blood and body fluid protocol as recommended by the manufacturer. After the removal of most proteins by digestion with proteinase K, the sample was applied to the QIAamp 96 plate. DNA was adsorbed onto the silica membrane during a brief centrifugation step, while any remaining protein, salt and other contaminants were completely removed by three consecutive washes. Membrane-bound DNA was then eluted in double deionized H_2_O or Tris–EDTA buffer. A final elution volume of 200 µl was used. Quantification of DNA was carried out using a PicoGreen DNA detection kit (Invitrogen). Calf thymus DNA (100 mg/ml; Sigma-Aldrich) was used as the standard. The concentration of DNA in the standard curve ranged from 0 to 100 ng/ml. Briefly, 20 ml of final DNA eluated was mixed with 1 ml of Tris-EDTA (10 mmol/l Tris–HCl, 1 mmol/l EDTA, pH 7.5) diluted with PicoGreen reagent. Fluorescence intensity was measured on an F-2000 spectrofluorometer (Molecular Devices) at excitation wavelength of 480 nm and an emission of 520 nm. Standard curve used to determine the levels of circulating DNA in the samples was established by the linear relationship between the known concentrations of calf thymus DNA (Sigma-Aldrich) and the corresponding fluorescence intensities.

### Cell Culture and Stimulation

RAW264.7 cells or peritoneal macrophages were cultured in Dulbecco’s modified Eagle’s medium (Invitrogen) supplemented with 10% fetal calf serum (Invitrogen) and 100 units/ml penicillin/streptomycin (Invitrogen) in a 5% CO_2_ incubator at 37°C. MBL (10 µg/ml) was incubated with ALD-DNA (ALD-DNA/MBL) for 1 h before stimulating RAW264.7 cells or peritoneal macrophages. RAW264.7 cells or peritoneal macrophages were treated with PBS, ALD-DNA (50 µg/ml) or ALD-DNA/MBL (50 µg/ml) for 24 h.

### ELISA Assay

The protein levels of TNF-α, MCP-1, IL-6 and IL-10 in the supernatants were assessed by ELISA assay with cytokine ELISA kits (eBioscience) according to the manufacturer’s instructions. A standard curve was generated using known amounts of the respective purified recombinant mouse cytokines or chemokines.

### Real-time PCR Analysis

Total RNA was extracted from cultured cells with TRIzol reagent (Invitrogen) according to the manufacturer’s instructions and the cDNA was synthesized with PrimeScript RT reagent kit (Takara Bio). The expression of the genes encoding IL-10, TNF-α, MCP-1 and IL-6 was quantified by real-time PCR using a Lightcycler480 and SYBR Green system (Roche Diagnostic Systems, Somerville, NJ), following the manufacturer’s protocol. The primer sequences used in this study are described previously [9].

### Flow Cytometric Analysis

Peritoneal macrophages were treated with PBS, ALD-DNA (50 µg/ml) or ALD-DNA/MBL (50 µg/ml) for 48 h. To assess the expression of activation and other biological markers on macrophages, flow cytometric analysis was performed with FITC-labeled anti-MHC class II, PE-labeled anti-CD80 and PE-labeled anti-CD86 (BD Biosciences). For intracellular analysis of cytokine production, T cells were pre-stimulated with 10 ng/ml phorbol myristate acetate and 1 µg/ml ionomycin in the presence of 10 µg/ml brefeldin A for 5 to 6 hours. T cells were first stained for APC-labeled anti-CD4 (BD Biosciences), then fixed, permeabilized, and stained for FITC-labeled anti-IFN-γ and PE-labeled anti-IL-17 and analyzed by flow cytometry. All flow cytometry data were acquired on a FACSCalibur (BD Biosciences) in CellQuest (BD Biosciences) and analyzed by FlowJo software (Tree Star, Ashland, OR).

### Cell Sorting

Murine renal tissues were surgical resected and dispersed in RPMI 1640 containing 5% FBS and 0.1% collagenase (Sigma-Aldrich) at 37°C for 30 min, followed by progressive sieving to obtain single-cell suspensions. To analyze gene expression in the renal macrophages, CD11b^+^/F4/80^high^ renal macrophages were sorted from nephritic single-cell suspensions using a FACSAria (BD Biosciences) with FITC-labeled anti-F4/80 and PE-labeled anti-CD11b (BD Biosciences). The purity of isolated cells was confirmed at 90%.

### Western Blot Analysis

Western blot analysis was performed as described previously [35]. Abs used here were anti-mouse iNOS (Santa Cruz Biotechnology), anti-mouse MBL (Santa Cruz Biotechnology), anti-IκB (Cell Signaling Technology), anti–β-actin (Santa Cruz Biotechnology), anti-CREB (Cell Signaling Technology), anti–phospho-CREB (Cell Signaling Technology), anti-ERK1/2 (Cell Signaling Technology), anti-phospho-ERK1/2 (Cell Signaling Technology), anti-p38 (Cell Signaling Technology), anti-phospho-p38 (Cell Signaling Technology), anti-JNK (Cell Signaling Technology), anti-phospho-JNK (Cell Signaling Technology), anti-goat IgG-HRP (Santa Cruz Biotechnology), anti-mouse IgG-HRP (Santa Cruz Biotechnology), and goat anti-rabbit IgG-HRP (Santa Cruz Biotechnology).

### Pathological Analysis

For histological analysis, murine kidney tissues were stained with H&E according to the manufacturer’s instructions. Fluorescent staining of cryosections was used for IC deposition analysis in the glomeruli. Sections were fixed in acetone for 10 min and incubated with FITC-conjugated goat anti-mouse IgG (H+L chain-specific) Abs (Sigma-Aldrich) for 30 min. Pictures were acquired with Nikon SCLIPSS TE2000-S microscope (Nikon, Melville, NY) equipped with ACT-1 software (Nikon; original magnification ×200).

### Statistical Analysis

Statistical significance was assessed using Student’s t-test or Mann–Whitney U-test unless otherwise noted, and data were given as mean ± SD unless otherwise noted. Statistical analyses of data were performed using the GraphPad Prism (version 4.0) statistical program. The statistical significance level was set as *p<0.05, **p<0.01, *** *p*<0.001.

## Results

### The Levels of MBL Decrease in Lupus Mice and are Negatively Correlated with SLE Disease

**Figure 1:**
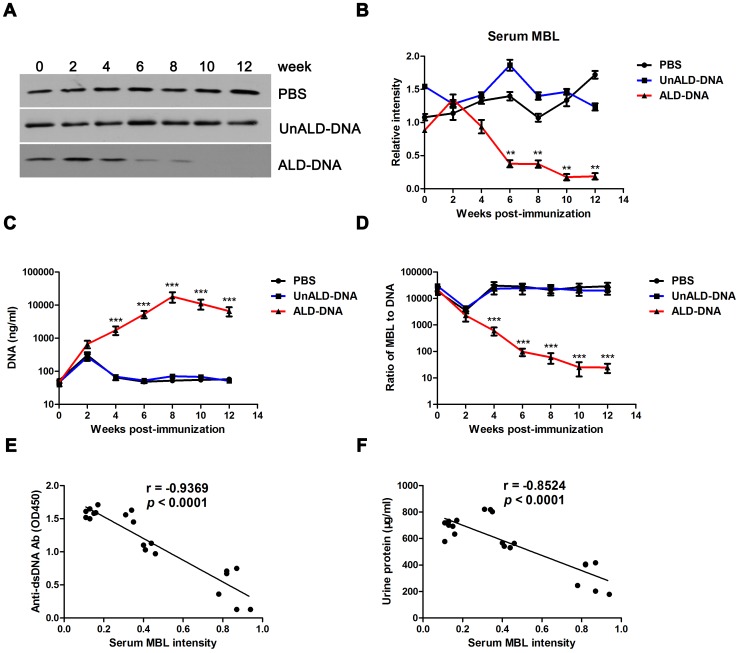
Serum MBL levels decrease in lupus mice and are negatively correlated with SLE disease. 6-week-old female BALB/c mice were immunized subcutaneously with ALD-DNA, UnALD-DNA, or PBS for 3 times in 4 weeks. n = 10. (A and B) Serum MBL levels in mice immunized with ALD-DNA, UnALD-DNA, or PBS were determined by western blot every 2 weeks and quantitative analysis of western blot of serum MBL levels was reflected as mean intensity. **p<0.01. n = 10. Data are representative of results obtained from 10 mice in each group. (C and D) The levels of serum circulating DNA were determined using a PicoGreen DNA detection kit and the ratios of MBL to DNA in SLE murine model and controls were presented. Data are means ± SD from 10 mice in each group. ***p<0.001. (E) The correlation between serum MBL and anti-dsDNA IgG level in SLE murine model (n = 20) was presented. Pearson correlation analysis was used to carry out the correlation study. r = −0.9369; *p*<0.001. (F) The correlation between serum MBL and urine protein level in SLE murine model (n = 20) was presented. Pearson correlation analysis was used to carry out the correlation study. r = −0.8524; *p*<0.001.

By means of immunization with ALD-DNA, we generated a murine model of SLE that developed high levels of anti-dsDNA antibodies (Figure S1A) and glomerulonephritis confirmed by urine protein quantification (Figure S1B), immune complex deposition assay (Figure S1C and S1D), and H&E staining of renal tissues (Figure S1E and S1F). To study whether MBL has a correlation to SLE disease, we first examined the levels of serum MBL in ALD-DNA–induced lupus mice and found that serum MBL levels increased slightly at week 2 but decreased sharply from week 4 (Figure 1A and 1B). From week 6, the levels of serum MBL in lupus mice were lower than those in controls. Enhanced circulating DNA levels were found in lupus mice (Figure 1C), which was consistent with our previous report [8], and the ratios of MBL to DNA were significantly lower in lupus mice than those in controls (Figure 1D). Furthermore, the levels of serum MBL were negatively correlated with the levels of anti-dsDNA antibodies and urine protein in SLE mice (Figure 1E and 1F). Taken together, these results indicate that MBL was insufficient in lupus mice.

### MBL Blunts ALD-DNA–induced Macrophage M2b Polarization

**Figure 2:**
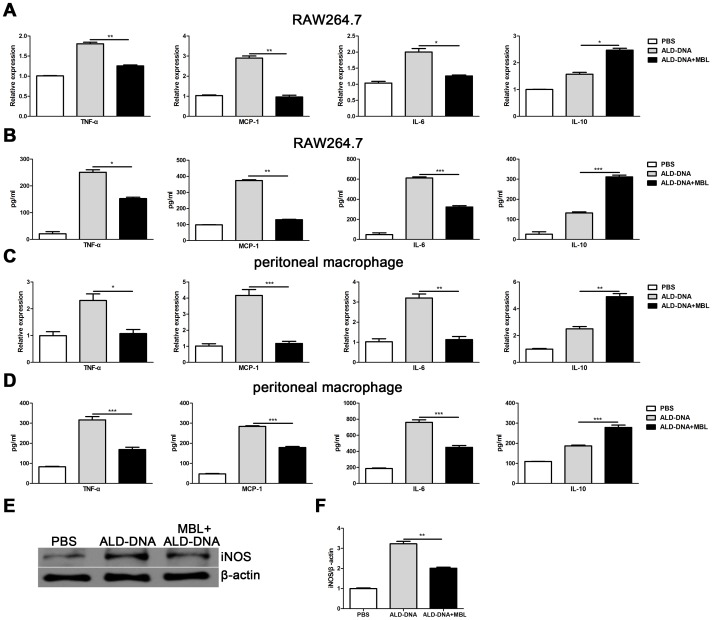
MBL modulates ALD-DNA–induced macrophage M2b polarization. ALD-DNA was pre-incubated with indicated mouse MBL for 2 h. RAW264.7 cells or peritoneal macrophages were cultured with PBS, ALD-DNA or ALD-DNA/MBL complexes for 24 h. (A and C) mRNA levels of TNF-α, MCP-1, IL-6 and IL-10 in RAW264.7 cells or peritoneal macrophages were analyzed by real-time PCR. (B and D) Protein levels of TNF-α, MCP-1, IL-6 and IL-10 in the supernatants of RAW264.7 cells or peritoneal macrophages were measured by ELISA. (E and F) RAW264.7 cells were cultured with PBS, ALD-DNA or ALD-DNA/MBL complexes for 24 h. Then cell lysates were prepared to measure the protein levels of iNOS by western blot analysis. All values are given relative to the expression level of the β-actin. Data are means ± SD of three independent experiments. *p<0.05; **p<0.01; ***p<0.001.

Our previous studies revealed that ALD-DNA could induce macrophage M2b polarization, which exhibited significantly enhanced expression of IL-10, TNF-α, IL-6, MCP-1, and inducible NO synthase (iNOS) and played a pivotal role in the development of ALD-DNA–induced SLE disease [7], [9]. To clarify whether MBL has any effect on the ALD-DNA–induced macrophage M2b polarization, we performed ELISA assay and real-time PCR to examine the expression of cytokines in mouse macrophage cell line RAW264.7 cells. We found that MBL could significantly suppress expression of the pro-inflammatory cytokines TNF-α, MCP-1 and IL-6 while greatly enhance expression of IL-10 at both mRNA and protein levels (Figure 2A and 2B). To examine whether the influence of MBL on the cytokine production of mouse primary macrophages is similar to its influence on those of RAW264.7 cells, ELISA assay and real-time PCR were performed to examine the expression of cytokines in peritoneal macrophages. Similar to what was observed in RAW264.7 cells, MBL treatment could suppress ALD-DNA–induced the expression of pro-inflammatory cytokines TNF-α, MCP-1 and IL-6 while significantly increase the expression of IL-10 in peritoneal macrophages (Figure 2C and 2D). Furthermore, we tested the protein expression of iNOS and found that MBL could suppress the level of iNOS induced by ALD-DNA (Figure 2E and 2F). Taken together, these results indicate that MBL treatment could modulate ALD-DNA–induced macrophage M2b polarization.

### Regulation of Macrophage M2b Polarization by MBL is Associated with Repression of MAPK and NF-κB Signaling Pathway while Increase of CREB Activation

**Figure 3:**
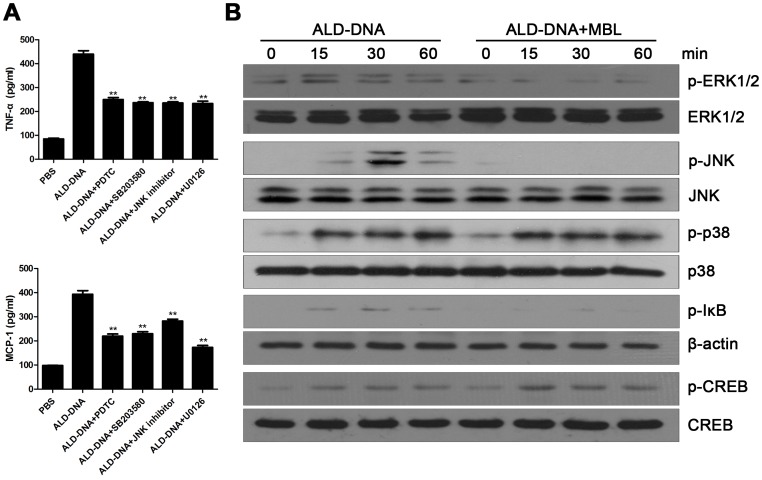
The signaling pathway of regulation of macrophage M2b polarization by MBL. (A) RAW264.7 cells were treated with a given inhibitor for 1 h before PBS or ALD-DNA stimulation and 24 h later the supernatants were collected for ELISA. (B) RAW264.7 cells were treated with ALD-DNA (50 µg/ml) or ALD-DNA (50 µg/ml) plus MBL (10 µg/ml) for the indicated times. The cells were harvested and β-actin, phosphorylated IκB, and the total and phosphorylated p38, ERK1/2, JNK and CREB were detected by western blot. Data are representative of results obtained in three independent experiments. **p<0.01.

As MBL could blunt macrophage M2b polarization, we next investigated whether MBL has any effect on the signaling pathway induced by ALD-DNA. We performed western blot and found that MBL could significantly down-regulate the phosphorylation of ERK1/2, JNK and IκB (Figure 3B), which was consistent with our previous reports that ALD-DNA could induce macrophage M2b polarization by activating the MAPK and NF-κB signaling [9](Figure 3A). Previous data indicate that C1q could increase the phosphorylation of the transcription factors cAMP response element-binding protein (CREB) and induce CRE driven gene expression, which was able to stimulate IL-10 production and modulate M2 macrophage-specific gene expression [36], [37]. Because MBL shared the similar structure and cell receptors with C1q [38], we investigated the phosphorylation of CREB and found that MBL could up-regulate the phosphorylation of CREB (Figure 3B). These results demonstrate that regulation of macrophage M2b polarization by MBL was associated with repression of MAPK and NF-κB signaling pathway while increase of CREB activation.

### MBL Treatment Blunts Macrophage M2b Polarization and Ameliorates Lupus Nephritis in ALD-DNA–induced SLE Mice

**Figure 4:**
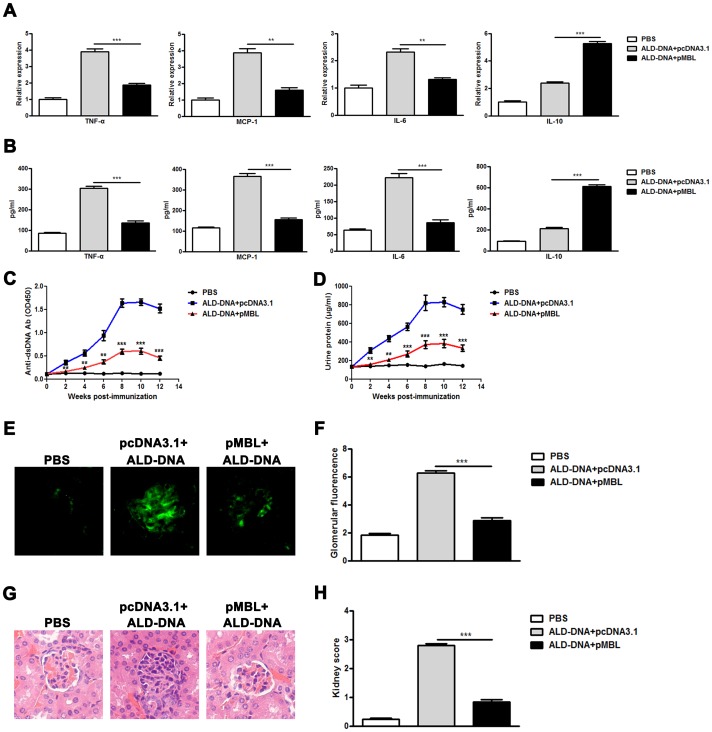
MBL treatment blunts macrophage M2b polarization and alleviates lupus nephritis. BALB/c mice were immunized subcutaneously with ALD-DNA (50 µg/mouse) or PBS for total 3 times in 4 weeks. Mice immunized with ALD-DNA were pre-treated intramuscularly with pMBL (100 µg/mice) or pcDNA3.1 (100 µg/mice), and injected every 2 weeks for total 5 times. (A) mRNA levels of TNF-α, MCP-1, IL-6 and IL-10 in renal macrophages purified from mice were evaluated by real-time PCR. Data are means ± SD of three independent experiments. **p<0.01; ***p<0.001. n = 6. (B) Levels of TNF-α, MCP-1, IL-6 and IL-10 in serum were detected by ELISA assay. ***p<0.001. n = 6. (C) Serum anti-dsDNA IgG levels of the mice were measured by ELISA assay every 2 weeks. **p<0.01; ***p<0.001. (D) Urine protein levels of the mice were assessed by BCA Protein Assay Kit every 2 weeks. **p<0.01; ***p<0.001. (E) The deposition of IgG-containing IC in glomeruli at week 12 after initial immunization. Imagines (×200) are representative of 10 mice in each group. (F) Mean glomerular fluorescence intensity (arbitrary units) was determined for IgG in ALD-DNA immunized lupus mice and control mice at week 12 after initial immunization. n = 10. ***p<0.001. (G) 12 weeks after initial immunization, nephritic pathological changes were shown by H&E staining of renal tissues surgical resected from the mice. Imagines (×200) are representative of 10 mice in each group. (H) The kidney score was assessed using paraffin sections stained with H&E. n = 10. ***p<0.001.

To evaluate the effect of MBL treatment in mice, mice immunized with ALD-DNA were treated with pMBL or pcDNA3.1. The levels of serum MBL were significantly increased and the levels of serum circulating DNA were notably decreased in the mice treated with pMBL as compared with those in the mice treated with pcDNA3.1 (Figure S2 and S3). Our previous study has revealed that macrophage M2b polarization played a pivotal role in the development of SLE disease. Because MBL could blunt macrophage M2b polarization induced by ALD-DNA *in vitro*, we further investigated whether MBL treatment could modulate macrophage polarization and lupus nephritis in ALD-DNA–induced lupus mice. Consistent with above results *in vitro*, MBL treatment could suppress the pro-inflammatory cytokine TNF-α, MCP-1 and IL-6 while increase the expression of IL-10 in the renal macrophages (Figure 4A) and in serum of lupus mice (Figure 4B). More importantly, notably reduced the levels of anti-dsDNA autoantibodies (Figure 4C), urine protein (Figure 4D), IC deposition (Figure 4E and 4F), renal pathology (Figure 4G), and kidney score (Figure 4H) were found in the pMBL-treated lupus mice as compared with those of pcDNA3.1-treated lupus mice. These results suggest that MBL treatment could ameliorate ALD-DNA–induced lupus nephritis.

### The Alleviation of SLE Disease by MBL Treatment might be Associated with Repression of the Ability of Macrophages to Polarize T Cells Toward Th1 and Th17 Cells

**Figure 5:**
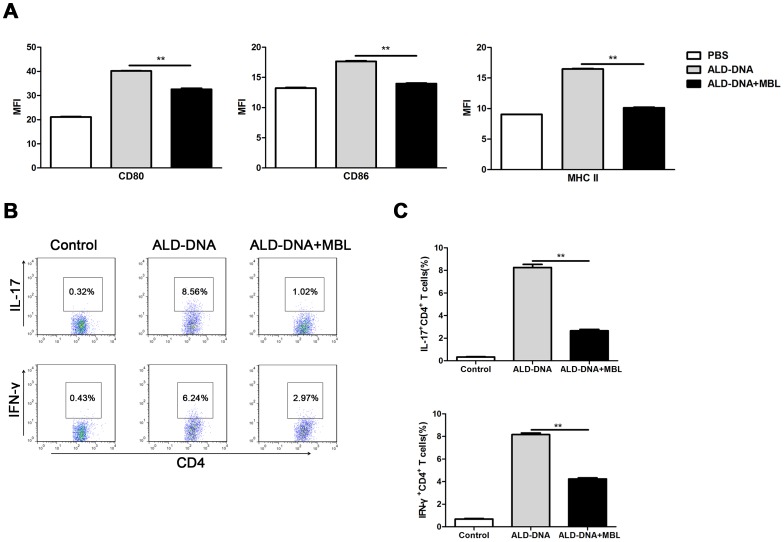
MBL alters the ability of ALD-DNA–stimulated macrophages to promote T cell differentiation. Peritoneal macrophages were incubated with PBS, ALD-DNA (50 µg/ml) or ALD-DNA (50 µg/ml) plus MBL (10 µg/ml) for 48 h. (A) The expression of MHC II, CD80 and CD86 was examined by flow cytometry and the levels of them were represented as mean fluorescent intensity (MFI). (B and C) These peritoneal macrophages and allogeneic T cells isolated from female BALB/c mice were cultured at a 1∶5 ratio in the presence of ALD-DNA (50 µg/ml) for 6 days. For intracellular analysis of cytokine production, T cells were pre-stimulated with 10 ng/ml phorbol myristate acetate and 1 µg/ml ionomycin in the presence of 10 µg/ml brefeldin A for 5 to 6 hours. Cells were then fixed, permeabilized, and stained for IFN-γ and IL-17 and analyzed by flow cytometry. Average of percentage of IFN-γ^+^ CD4^+^ or IL-17^+^CD4^+^ T cells was presented in the bar charts. Data are means ± SD of three independent experiments. **p<0.01.

T cell differentiation plays an important role in the development of SLE disease. Activated macrophages with elevated expression of activation markers have the acquisition of antigen-presenting features, which lead to efficient Th1 and Th17 responses [39], [40]. MBL could regulate ALD-DNA*–*induced macrophage activation and polarization, so we next investigated whether the regulation of T cell differentiation will occur in the development of modulation of ALD-DNA*–*induced immune responses by MBL. We applied flow cytometry and found that MBL could decrease the expression of MHC II, CD80 and CD86 (Figure 5A). So we suggested that MBL could modulate the differentiation of T cells. To determine whether MBL influences the ability of macrophages to modulate the differentiation of T cells, we cultured peritoneal macrophages with PBS, ALD-DNA or ALD-DNA plus MBL for 48 h and then used them as APCs to stimulate the allogeneic naïve T cells for 6 days. It was found that there were significantly less IL-17 and IFN-γ production by CD4^+^ T cells incubated with ALD-DNA/MBL–stimulated peritoneal macrophages than those incubated with ALD-DNA–stimulated peritoneal macrophages (Figure 5B and 5C). Further study showed that IL-17^+^CD4^+^ and IFN-γ^+^CD4^+^ T cells in lupus mice injected with ALD-DNA/MBL–treated macrophages were decreased significantly as compared with those in lupus mice injected with ALD-DNA–treated macrophages (Figure S4A and S4B). These results suggest that MBL suppressed the ability of ALD-DNA–stimulated macrophages to promote the differentiation of CD4^+^ T cells into the Th1 and Th17 phenotypes, which might be involved in the alleviation of ALD-DNA–induced SLE disease by MBL.

## Discussion

It has been reported that MBL deficiency or low serum MBL levels caused by polymorphisms in the structural portion or promoter region of the MBL gene were observed in SLE patients [15], [27], [28]. However it remains unclear whether it contributes to the initiation and progression of SLE, especially the innate and adaptive immunity that occur in the development of autoimmune responses. Data presented in this study showed that the levels of MBL decreased in lupus mice and were negatively correlated with SLE disease. MBL blunted macrophage M2b polarization and suppressed the ability of macrophages to promote the differentiation of T cells. Furthermore, MBL treatment ameliorated ALD-DNA–induced lupus nephritis by reducing anti-dsDNA antibodies production and IC deposition.

It is now well established that exorbitant cell apoptosis has been found to occur frequently in SLE patients and lupus mice, which is caused by various factors including genetic susceptibility and environmental factors [41], [42]. Impaired clearance ability for apoptotic cells and cellular debris, including defects of macrophages and deficiency of serum factors such as complement, DNase I, pentraxins and IgM, may explain accumulation of self nuclear antigens released from noningested apoptotic cells [43]–[45]. Nuclear antigens including self-dsDNA released from these excessive apoptotic cells have been reported to contribute to the activation of pathological immune responses and the production of autoantibody, which lead to IC deposition and organ damage [46].

MBL is one member of complement components. In addition to the removal of microorganisms by activating the complement system, MBL could mediate the recognition and the clearance of modified self cells, such as apoptotic cells and cellular debris, which is important for maintaining tissue homeostasis and avoiding autoimmune diseases such as SLE [22]–[25]. Previous studies have demonstrated that MBL deficiency or low serum MBL levels were observed in SLE patients [15], [27], [28], but whether MBL is involved in the development of SLE remains unclear. In our previous studies, we generated a novel murine model of SLE by immunizing the syngeneic female BALB/c mice with ALD-DNA, which developed high titers of anti-dsDNA antibodies, proteinuria, and glomerular nephritis that closely resembled human SLE [6]–[8]. The levels of serum MBL in lupus mice induced by ALD-DNA decreased and were negatively correlated with SLE disease, indicating that serum MBL was insufficient in SLE disease, which was consistent with previous reports on the serum MBL levels in SLE patients and MRL-lpr lupus mice [15], [27], [28], [47]. Our results revealed that MBL treatment could enhance the clearance of DNA (Figure S3), indicating that MBL might play a protective role in the development of SLE. Previous studies indicated that MBL could enhance the clearance of modified cells and cells debris by the interaction with its receptors on the cell surface, such as C1q phagocytic receptor C1qRp (CD93), cC1qR (CRT) and CR1 (CD35) [19]–[25]. The involved receptor(s) of MBL in the process of DNA clearance remains to be further studied. In addition to the enhancement of DNA clearance, MBL was proved to bind to immunoglobulins and might facilitate the clearance of circulating immune complexes [48], [49]. The clearance of DNA and immune complexes mediated by MBL might exhaust the serum MBL. Besides, it was observed that there was a strong inverse correlation between anti-MBL autoantibodies and serum MBL levels [50]. The precise mechanism of decrease of serum MBL in ALD-DNA–induced lupus mice remains to be further studied. Furthermore, our study showed that MBL supplement *in vivo* could reduce the levels of anti-dsDNA autoantibodies, urine protein, IC deposition, renal pathology and kidney score. These data provide evidence to support the protective role of MBL in SLE disease.

Functional macrophage polarization represented different extremes of a continuum ranging from M1, M2a and M2b to M2c [9]. M1 polarization, driven by IFN-γ and LPS, typically acquires fortified cytotoxic and antitumoral properties; M2a polarization, induced by IL-4 and IL-13, and M2b polarization, induced by combined immune complexes with TLR or IL-1R agonists, exert immunoregulatory functions and drive type II responses, whereas M2c polarization, induced by IL-10, gains immunosuppression and tissue-remodeling activities. Our previous studies revealed that ALD-DNA could induce macrophage M2b polarization, which exhibited enhanced IL-10, TNF-α, IL-6, MCP-1 and iNOS [9]. Recent studies suggest that MBL could modulate macrophage-mediated inflammatory responses [29], [30]. To study whether MBL has any influence on the immune responses induced by ALD-DNA, we examined cytokine production and found that MBL treatment leaded to the suppression of ALD-DNA–induced pro-inflammatory cytokines TNF-α, MCP-1 and IL-6, and an increase of anti-inflammatory cytokine IL-10 in macrophages, indicating that ALD-DNA/MBL treatment could turn M2b macrophages to an anti-inflammatory phenotype. Further studies are needed to investigate the definitive anti-inflammatory phenotype induced by ALD-DNA/MBL. Importantly, we found that MBL could significantly down-regulate the phosphorylation of ERK1/2, JNK and IκB induced by ALD-DNA, which was consistent with our previous reports that MAPK and NF-κB signaling played an important role in ALD-DNA–induced macrophage M2b polarization [9]. A previous study has reported that C1q could increase the phosphorylation of CREB, which could induce CRE driven IL-10 production and modulate M2 macrophage-specific gene expression [36], [37]. Because of the similar structure and cell receptors that MBL and C1q shared [38], we examined the phosphorylation of CREB and found MBL could up-regulate the phosphorylation of CREB, which might provide an interpretation of an increase of IL-10 in macrophages cultured with ALD-DNA/MBL. Apart from these signaling pathways, whether ALD-DNA/MBL modulates other signaling pathways remains to be investigated.

As antigen-presenting cells, activated macrophages can differentiate naïve T cells into Th1 cells and Th17 cells, which play a pivotal role in the development of SLE disease [51]–[53]. In this study, we found that MBL treatment decreased the expression of MHC II, CD80 and CD86, and leaded to the suppression of the ability of ALD-DNA–stimulated macrophages to promote the differentiation of CD4^+^ T cells into the Th1 and Th17 phenotypes *in vitro* and *in vivo*, which might be involved in the alleviation of SLE disease by MBL. These data indicate that MBL played an important role in the regulation of ALD-DNA–induced immune responses and would further our understanding of the regulatory function of MBL in the development of SLE.

In conclusion, our results presented in this study showed that the levels of serum MBL significantly decreased in lupus mice and were negatively correlated with SLE disease. MBL could blunt macrophage M2b polarization induced by ALD-DNA and suppress the ability of macrophages to promote T cell differentiation. MBL supplement *in vivo* could ameliorate ALD-DNA–induced lupus nephritis. Our findings provide a novel mechanism to understand the role of MBL in the pathogenesis of SLE and indicate that MBL might be a new candidate for modulating immune responses induced by DNA. So these results would open a new avenue for developing novel therapeutic approaches for DNA-induced autoimmune diseases such as SLE.

## Supporting Information

Figure S1 **ALD-DNA immunization induces high levels of anti-dsDNA antibody and lupus nephritis.** 6-week-old female BALB/c mice were immunized subcutaneously with ALD-DNA, UnALD-DNA, or PBS for total 3 times in 4 weeks. n = 10. (A) Serum anti-dsDNA IgG levels were measured by ELISA assay every 2 weeks after initial immunization. Data are means ± SD from 10 mice in each group. ***p<0.001. (B) Urine protein levels of the mice were assessed by BCA Protein Assay Kit every 2 weeks. Data are means ± SD from 10 mice in each group. ***p<0.001. (C) Glomerular immune complex deposition was detected by direct immunofluorescence for IgG in frozen kidney section from ALD-DNA-immunized lupus mice or control mice. Representative images (magnification×200) of 10 mice are shown for each group. (D) Mean glomerular fluorescence intensity (arbitrary units) was determined for IgG in ALD-DNA-immunized lupus mice and control mice. ***p<0.001. n = 10. (E) 12 weeks after initial immunization, nephritic pathology was evaluated by H&E staining of renal tissues. Imagines (magnification×200) are representative of at least 10 mice in each group. (F) The kidney score was assessed using paraffin sections stained with H&E. ***p<0.001.
(TIF)

Figure S2 **pMBL treatment significantly increases the serum MBL levels.** Mice immunized with ALD-DNA were treated intramuscularly with pMBL (100 µg/mice) or pcDNA3.1 (100 µg/mice), and injected every 2 weeks for total 5 times. The levels of serum MBL were detected by western blot every 2 weeks. Data are means ± SD from 10 mice in each group.
(TIF)

Figure S3 **MBL treatment enhances the clearance of DNA** ***in vitro*** **and** ***in*** **** ***vivo*** **.** ALD-DNA labeled with Alexa Fluor 488 (AF488-ALD-DNA) was incubated with MBL at 37°C for 2 h. And then RAW264.7 cells were cultured with PBS, AF488-ALD-DNA or AF488-ALD-DNA/MBL complexes for 30 minutes. (A and B) The intracellular AF488-ALD-DNA was determined by flow cytometry. ***p<0.001. (C) Mice immunized with ALD-DNA were treated intramuscularly with pMBL (100 µg/mice) or pcDNA3.1 (100 µg/mice), and injected every 2 weeks for total 5 times. 12 weeks after initial immunization, the levels of serum DNA in lupus mice treated with pMBL or pcDNA3.1 and control mice were detected using a PicoGreen DNA detection kit. Data are means ± SD from 10 mice in each group. *p<0.05.
(TIF)

Figure S4 **MBL suppresses the ability of macrophages to promote T cell differentiation** ***in vivo*** **.** For adoptive transfer of macrophages, we first treated peritoneal macrophages with ALD-DNA/MBL or ALD-DNA alone for 48 h. And then ALD-DNA–immunized mice were injected i.v with these macrophages (2.5×10^6^ cells/mouse) at weeks 0, 2 and 4 after the initial immunization for a total of three times. (A and B) 8 weeks after initial immunization, splenocytes were collected and the levels of Th1 and Th17 cells were analyzed by flow cytometry. Data are means ± SD from 10 mice in each group. **p<0.01.
(TIF)
