# Supplementary figures and images for: Mannose-Binding Lectin Blunts Macrophage Polarization and Ameliorates Lupus Nephritis
Source: PLoS One. 2013 Apr 23;8(4):e62465. doi: 10.1371/journal.pone.0062465 (PMC3633861; doi:10.1371/journal.pone.0062465)

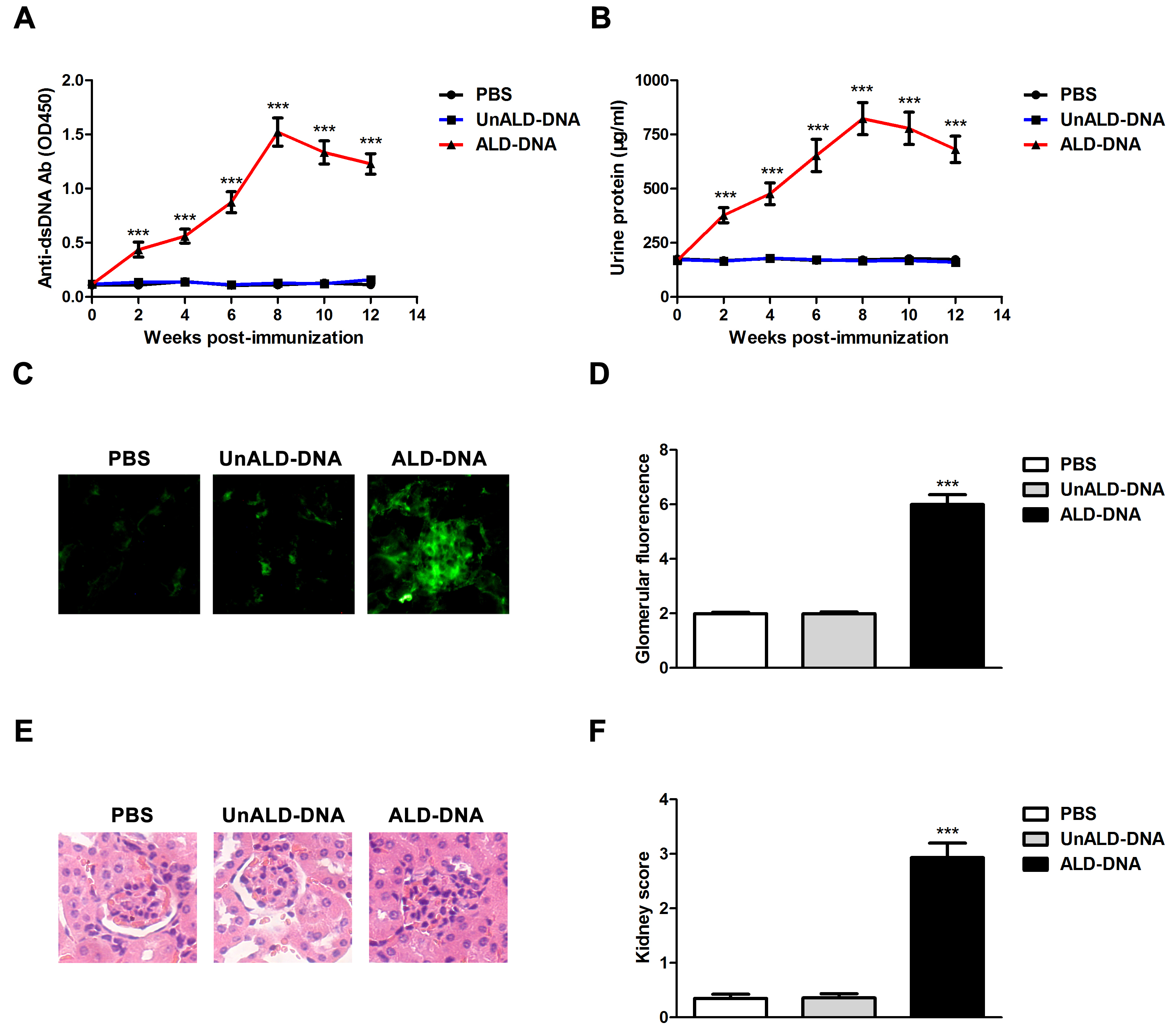

Supplement: Figure S1 — ALD-DNA immunization induces high levels of anti-dsDNA antibody and lupus nephritis. 6-week-old female BALB/c mice were immunized subcutaneously with ALD-DNA, UnALD-DNA, or PBS for total 3 times in 4 weeks. n = 10. (A) Serum anti-dsDNA IgG levels were measured by ELISA assay every 2 weeks after initial immunization. Data are means ± SD from 10 mice in each group. ***p<0.001. (B) Urine protein levels of the mice were assessed by BCA Protein Assay Kit every 2 weeks. Data are means ± SD from 10 mice in each group. ***p<0.001. (C) Glomerular immune complex deposition was detected by direct immunofluorescence for IgG in frozen kidney section from ALD-DNA-immunized lupus mice or control mice. Representative images (magnification×200) of 10 mice are shown for each group. (D) Mean glomerular fluorescence intensity (arbitrary units) was determined for IgG in ALD-DNA-immunized lupus mice and control mice. ***p<0.001. n = 10. (E) 12 weeks after initial immunization, nephritic pathology was evaluated by H&E staining of renal tissues. Imagines (magnification×200) are representative of at least 10 mice in each group. (F) The kidney score was assessed using paraffin sections stained with H&E. ***p<0.001. (TIF) [file pone.0062465.s001.tif]

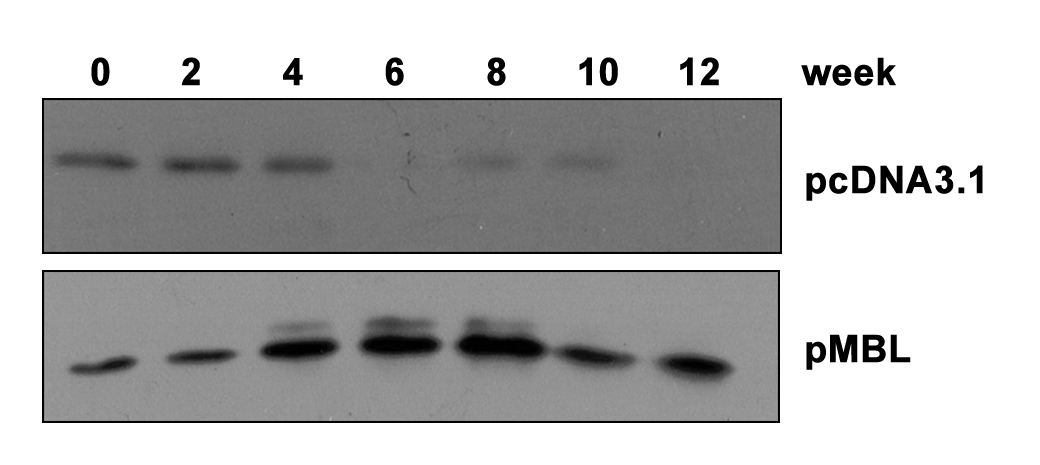

Supplement: Figure S2 — pMBL treatment significantly increases the serum MBL levels. Mice immunized with ALD-DNA were treated intramuscularly with pMBL (100 µg/mice) or pcDNA3.1 (100 µg/mice), and injected every 2 weeks for total 5 times. The levels of serum MBL were detected by western blot every 2 weeks. Data are means ± SD from 10 mice in each group. (TIF) [file pone.0062465.s002.tif]

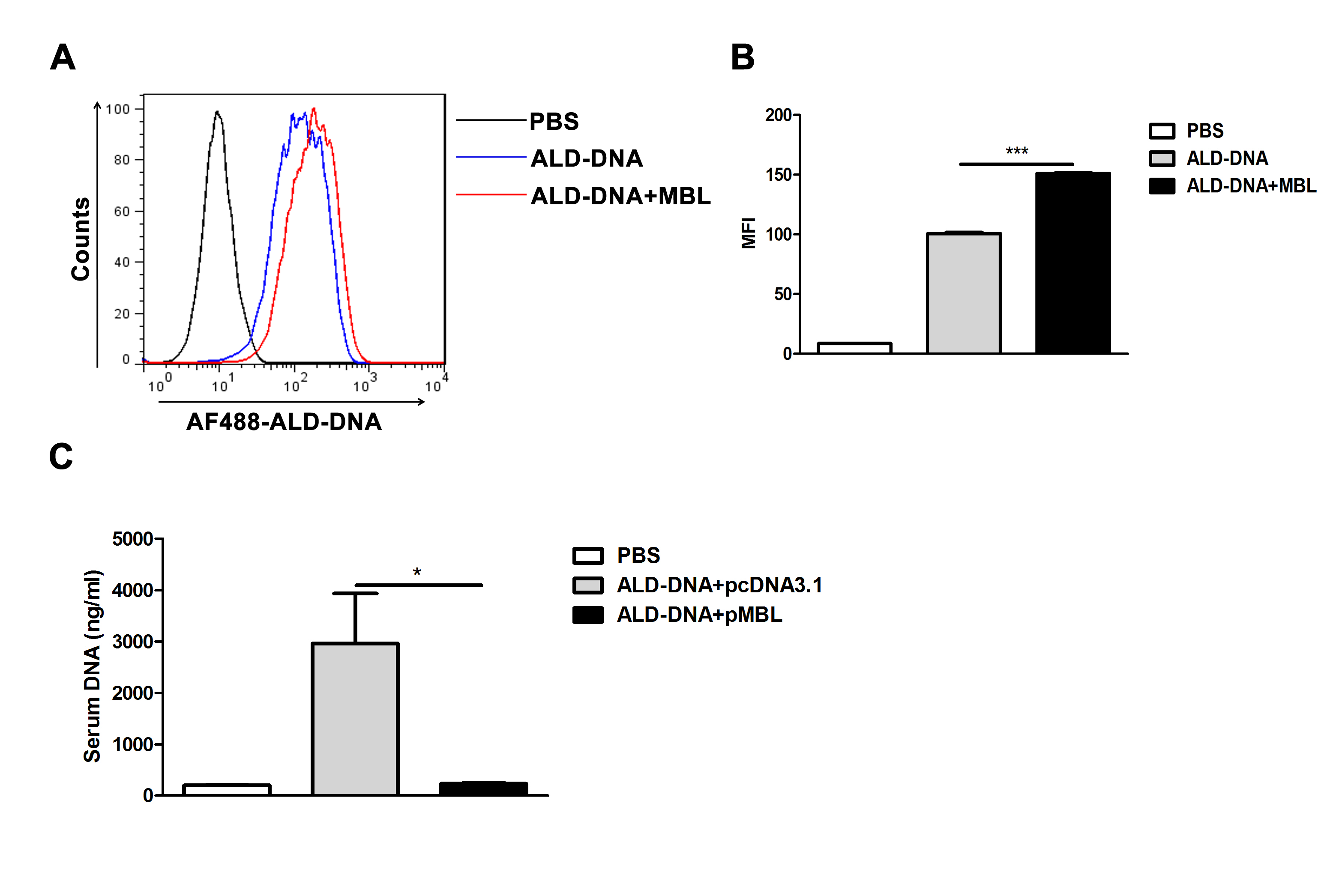

Supplement: Figure S3 — MBL treatment enhances the clearance of DNA in vitro and in vivo . ALD-DNA labeled with Alexa Fluor 488 (AF488-ALD-DNA) was incubated with MBL at 37°C for 2 h. And then RAW264.7 cells were cultured with PBS, AF488-ALD-DNA or AF488-ALD-DNA/MBL complexes for 30 minutes. (A and B) The intracellular AF488-ALD-DNA was determined by flow cytometry. ***p<0.001. (C) Mice immunized with ALD-DNA were treated intramuscularly with pMBL (100 µg/mice) or pcDNA3.1 (100 µg/mice), and injected every 2 weeks for total 5 times. 12 weeks after initial immunization, the levels of serum DNA in lupus mice treated with pMBL or pcDNA3.1 and control mice were detected using a PicoGreen DNA detection kit. Data are means ± SD from 10 mice in each group. *p<0.05. (TIF) [file pone.0062465.s003.tif]

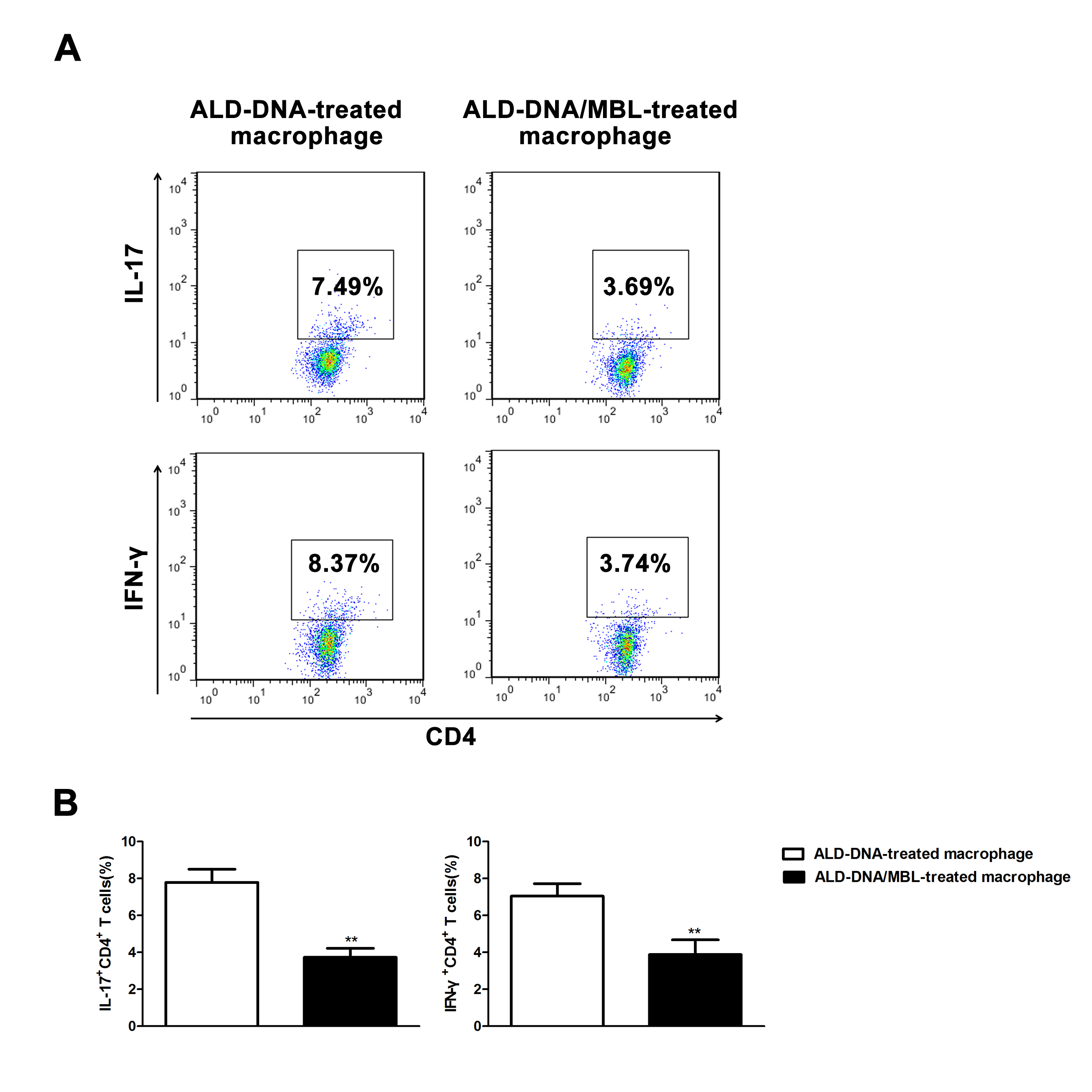

Supplement: Figure S4 — MBL suppresses the ability of macrophages to promote T cell differentiation in vivo . For adoptive transfer of macrophages, we first treated peritoneal macrophages with ALD-DNA/MBL or ALD-DNA alone for 48 h. And then ALD-DNA–immunized mice were injected i.v with these macrophages (2.5×10^6^ cells/mouse) at weeks 0, 2 and 4 after the initial immunization for a total of three times. (A and B) 8 weeks after initial immunization, splenocytes were collected and the levels of Th1 and Th17 cells were analyzed by flow cytometry. Data are means ± SD from 10 mice in each group. **p<0.01. (TIF) [file pone.0062465.s004.tif]
